# T41. MODIFIED COGNITIVE BEHAVIORAL THERAPY FOR ELDERLY PATIENTS WITH SCHIZOPHRENIA: A RANDOMIZED, CONTROLLED PILOT TRIAL

**DOI:** 10.1093/schbul/sby016.317

**Published:** 2018-04-01

**Authors:** Bernhard Müller, Christian Kärgel, Magdalena Horacek, Ute Darrelmann, Anja Heger, Daniela Kariofillis, Beate Nentwich, Christoph Meisner, Claudia Ose, Svenja Unsöld, Jens Wiltfang, Stefan Klingberg, Gudrun Sartory

**Affiliations:** 1 University of Duisburg-Essen; 2 LWL-University Hospital Bochum; 3 University of Bonn; 4 University of Wuppertal; 5 University of Tübingen; 6 University of Göttingen

## Abstract

**Background:**

An European society growing older sets the need to explore treatment options for elderly patients. Here we aimed to gather evidence on the effectiveness of a modified cognitive behavioural therapy (mCBT) in elderly schizophrenia patients as compared to treatment as usual (TAU).

**Methods:**

43 schizophrenia patients > 55y (mean 60 y), were assessed in a randomized, single blind controlled pilot trial with parallel groups: TAU+mCBT vs. TAU and intention to treat (ITT) last observation carried forward (LOCF) analysis. Subjects were recruited in Germany among in- and outpatients. MCBT comprised 30 sessions in 9 month, and a 6 month follow-up including a) physical and social activation, b) problem solving c) social skills. Primary outcomes were pre/post change in either PANSS total score, UPSA-brief score or Calgary Depression Scale for Schizophrenia.

**Results:**

From 43 patients, 40 were randomized. 15 mCBT and 16 TAU-patients comprised the ITT sample. None of the primary outcome measures reached significance. When assessing effect sizes we found little pre-/post change in PANSS total score (d=0.14) and the UPSA-brief (d=0.14). Depression symptoms however improved with treatment (pre/post d=0.75 and pre/FU d=0.52). Among secondary outcomes, global assessment of functioning significantly improved in the mCBT-group pre/post (d=.074) and pre/follow-up (d=.080).

**Discussion:**

Our results provide evidence for the feasibility of a sufficiently powered phase-III study targeting depressive symptoms and global functioning in long-term treated elderly schizophrenia patients. A history of about twenty years of pharmacological treatment does not imply there is no room for improvement in depression and global functioning following a modified cognitive behavioral therapy in this specific patient group. Supported by the German Federal Ministry of Education and Research (BMBF-01GV0909); ICTRP/DRKS: DRKS00003623.

